# Cell type-specific modulation of healthspan by Forkhead family transcription factors in the nervous system

**DOI:** 10.1073/pnas.2011491118

**Published:** 2021-02-15

**Authors:** Ekin Bolukbasi, Nathaniel S. Woodling, Dobril K. Ivanov, Jennifer Adcott, Andrea Foley, Arjunan Rajasingam, Lauren M. Gittings, Benjamin Aleyakpo, Teresa Niccoli, Janet M. Thornton, Linda Partridge

**Affiliations:** ^a^Institute of Healthy Ageing, University College London, London WC1E 6BT, United Kingdom;; ^b^Department of Genetics, Evolution and Environment, University College London, London WC1E 6BT, United Kingdom;; ^c^European Bioinformatics Institute, European Molecular Biology Laboratory, Cambridge CB10 1SD, United Kingdom;; ^d^UK Dementia Research Institute, Cardiff University, Cardiff CF24 4HQ, United Kingdom;; ^e^Department of Biological Mechanisms of Ageing, Max Planck Institute for Biology of Ageing, 50931 Cologne, Germany

**Keywords:** aging, Alzheimer’s disease, glia, neurons, transcription factors

## Abstract

Reduced activity of insulin/insulin-like growth factor signaling (IIS) increases healthy lifespan among diverse animal species. Downstream of IIS, multiple evolutionarily conserved transcription factors (TFs) are required; however, distinct TFs are likely responsible for these effects in different tissues. Here we have asked which TFs can extend healthy lifespan within distinct cell types of the adult nervous system in *Drosophila*. Starting from published single-cell transcriptomic data, we report that *forkhead* (FKH) is endogenously expressed in neurons, whereas *forkhead-box-O* (FOXO) is expressed in glial cells. Accordingly, we find that neuronal FKH and glial FOXO exert independent prolongevity effects. We have further explored the role of neuronal FKH in a model of Alzheimer’s disease-associated neuronal dysfunction, where we find that increased neuronal FKH preserves behavioral function and reduces ubiquitinated protein aggregation. Finally, using transcriptomic profiling, we identify *Atg17*, a member of the Atg1 autophagy initiation family, as one FKH-dependent target whose neuronal overexpression is sufficient to extend healthy lifespan. Taken together, our results underscore the importance of cell type-specific mapping of TF activity to preserve healthy function with age.

## Significance

Aging is the main risk factor for the costliest diseases in today’s world. However, significant gaps remain in understanding how different cell types modulate this most common physiological process. Here, we use published single-cell gene expression data to map the prolongevity roles of two evolutionarily conserved *Drosophila* transcription factors, FKH and FOXO, onto either neuronal or glial cells. We then demonstrate that neuronal FKH preserves healthy function even under stress. Finally, we identify an autophagy-related gene as one of FKH’s downstream prolongevity effectors. Our results exemplify tapping into publicly available gene expression datasets to extract physiological insights, and highlight the need to shift away from organism-wide approaches and toward cell type-specific strategies to obtain meaningful insights in aging research.

Metazoan life is defined by its diversity of cell types, each type maintaining its identity and reacting to its environment through the cell type-specific action of transcription factors (TFs) that regulate appropriate gene expression patterns. In some cases, a single stimulus acts through distinct TFs in different cell types to produce divergent effects. For instance, the insulin/insulin-like growth factor (IGF) signaling (IIS) pathway coordinates nutrition with growth and metabolism across tissues throughout the animal kingdom. Insulin and insulin-like ligands promote glucose uptake and glycogen synthesis in myocytes, reduce gluconeogenesis and promote de novo lipogenesis in hepatocytes, and reduce lipolysis in adipocytes; each cell type responds through partially overlapping signaling cascades that exert their long-term effects through distinct combinations of TFs (1).

While IIS activity is indispensable for proper development and metabolism, reduced IIS in adult organisms can extend healthy lifespan in diverse species (2, 3). These findings have prompted the hypothesis that IIS is a canonical example of antagonistic pleiotropy, that is, a biological process that has been evolutionarily favored for its role in youth despite its detrimental effects in aging (4). Consistent with this hypothesis, multiple interventions that inhibit IIS, including reduced levels of insulin-like ligands (5, 6), reduced signaling through the insulin receptor and its substrates (7–11), and inhibition of the effector kinases PI3K (12) and RAS (13), lead to increased healthy lifespan, stress resistance, and preserved function with age in multiple species. In humans as well, gene variants that reduce the activity of the IGF1 receptor are enriched in centenarians (14). With the predicted continued rise in the proportion of elderly individuals across industrialized nations (15), a more complete understanding of how IIS modulates aging through diverse cell types is needed to guide effective therapeutic interventions for the diseases of aging.

Studies at the level of the whole organism, and an increasing number at the level of individual tissues, have revealed that transcriptional regulation is essential for the IIS pathway to mediate its effects on lifespan. Inhibition of Forkhead-box family TFs, particularly FOXO family members, are the best characterized lifespan-limiting effects of IIS activity (12, 16, 17). Consistent with a prolongevity role for FOXO family members in humans, single-nucleotide polymorphisms in the *FOXO3A* gene are associated with longevity in large-scale genome-wide association studies (18, 19). In addition to FOXO, multiple additional TFs within the IIS pathway modulate healthy lifespan at the level of the whole organism, including ETS family members (20) and FOXA family members (21). While FOXA family members have been less extensively studied than FOXO downstream of IIS, recent work has shown that IIS activity results in phosphorylation of the *Drosophila* FOXA ortholog Forkhead (FKH) and that FKH can biochemically interact with both AKT and TOR kinases (21), identifying FKH as a key transcriptional player within the IIS/TOR signaling network.

Remarkably, modulation of TFs downstream of IIS can extend lifespan even when restricted to specific tissues (reviewed in ref. 22). Studies in *Drosophila* have demonstrated increased lifespan for flies with increased FOXO expression in muscle, gut, and/or adipose tissues (17, 23, 24), with distinct FOXO-dependent and -independent prolongevity effects in different tissues (25). Indeed, within the gut, increased expression of FKH in differentiated intestinal cells is sufficient to extend lifespan (21). These studies suggest that distinct Forkhead family TFs may have specific roles in modulating healthy lifespan in individual cell types.

Among organ systems, the effects of aging on the nervous system present particularly important challenges to modern medicine. The increasing prevalence of the neurodegenerative diseases of aging is one of the most costly consequences of the rapidly aging demographic of human populations (26). In some developed countries, Alzheimer’s disease and related causes of dementia have now become the leading cause of death (27). Moreover, IIS activity in the nervous system can directly modulate healthy lifespan and nervous system function with age. In mice, IGF1 receptor deletion extends lifespan when restricted to the nervous system using the *nestin-Cre* driver line, which expresses in both neurons and glia (the nonneuronal cells of the nervous system) (28). In flies, IIS inhibition in either neurons (29, 30) or glia (31) alone is sufficient to extend healthy lifespan, with neuron-specific interventions able to preserve youthful electrical transmission in neuronal circuits with age (32). However, studies to date have not identified which of the TFs downstream of IIS can extend healthy lifespan in the nervous system. Paradoxically, increased FOXO expression in neurons dramatically shortens *Drosophila* lifespan (17, 20), suggesting that other TFs may play more protective roles in neurons and/or glia.

To this end, we hypothesize here that accurate mapping of the TFs endogenously expressed in different cell types could be indicative of their potential to modulate lifespan in a cell type-specific manner. Drawing on recent single-cell RNA-sequencing (RNA-seq) profiling of the adult *Drosophila* brain (33), we show here that FOXO is endogenously expressed in glia but not neurons, whereas FKH is expressed in neurons but not glia. Consistent with these expression patterns, we show that increased expression of FOXO in glia and FKH in neurons can extend healthy lifespan. Moreover, we find that neuronal FKH can preserve function in a *Drosophila* model of Alzheimer’s disease-related amyloid-beta (Aβ) toxicity. Finally, using transcriptomic profiling, we identify increased expression of *Atg17*, an essential component of the Atg1 autophagy initiation complex, as one of the beneficial effects downstream of FKH in neurons in response to reduced IIS.

## Results

### FKH, but Not FOXO, Can Extend Lifespan in *Drosophila* Neurons.

**Fig. 1.**
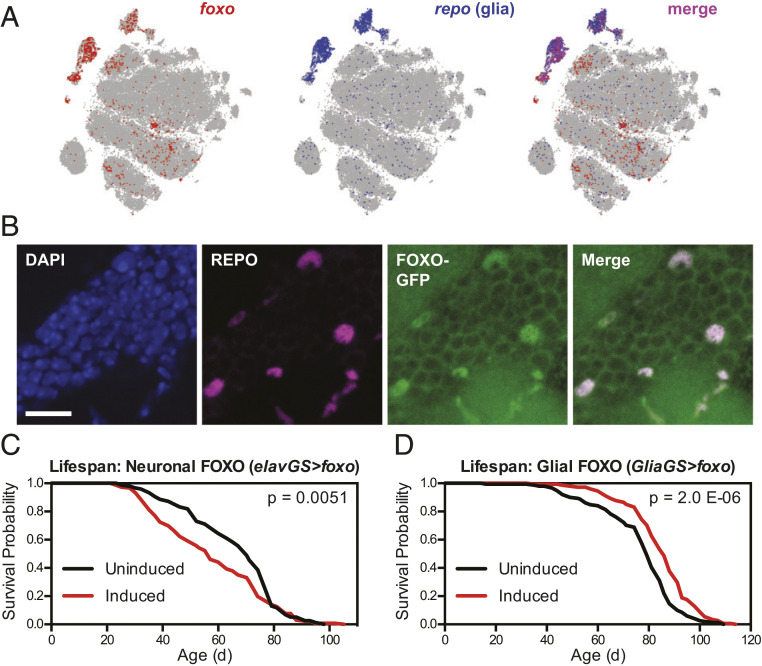
Overexpression of *foxo* in glia, but not neurons, extends lifespan. (*A*) Images from the SCope database (33) show mRNA expression of *foxo* largely in *repo*-expressing (glial) cell populations in the *Drosophila* brain. (*B*) Immunofluorescence images from the cell body layer adjacent to the olfactory bulb in the central brain show FOXO-GFP expression overlapping with REPO-positive glial cells in *w*^*Dah*^*;foxo-GFP* flies. (Scale bar, 10 μm.) (*C* and *D*) Survival curves show (*C*) shortened lifespan for *w*^*Dah*^*;;elav-GS/UAS-foxo* flies and (*D*) extended lifespan for *w*^*Dah*^*;;GSG3285-1/UAS-foxo* flies treated with 200 μM RU-486 from 2 d of age compared with sibling flies of the same genotype treated with vehicle control food. For all survival experiments, *n* > 140 deaths were counted per condition; *P* values are from log-rank tests between groups.

**Fig. 2.**
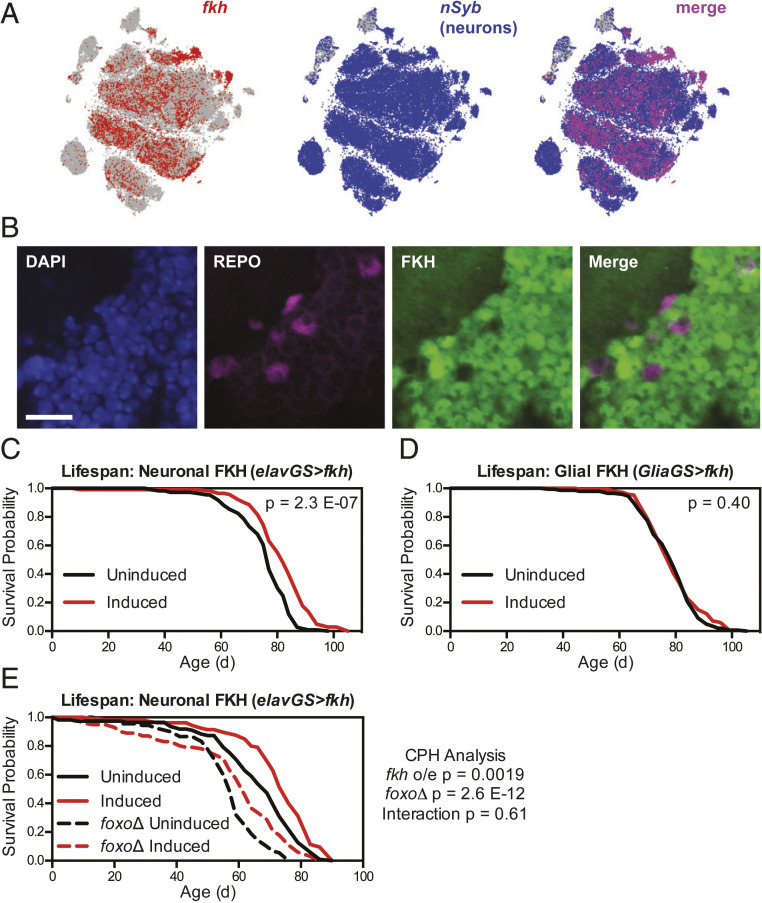
Overexpression of *fkh* in neurons, but not glia, extends lifespan independent of FOXO. (*A*) Images from the SCope database (33) show mRNA expression of *fkh* largely in *nSyb*-expressing (neuronal) cell populations in the *Drosophila* brain. (*B*) Immunofluorescence images from the cell body layer of the central brain show FKH expression in REPO-negative (neuronal) cells in *w*^*Dah*^ flies. (Scale bar, 10 μm.) (*C*–*E*) Survival curves show (*C*) extended lifespan for *w*^*Dah*^*;UAS-fkh/+;elav-GS/+* flies, (*D*) no change in lifespan for *w*^*Dah*^*;UAS-fkh/+;GSG3285-1/+* flies, and (*E*) extended lifespan for both *w*^*Dah*^*;UAS-fkh/+;elav-GS/+* and *w*^*Dah*^*;UAS-fkh/+;elav-GS, foxo*Δ*/foxo*Δ flies treated with 200 μM RU-486 from 2 d of age, with no significant interaction between *fkh* overexpression and *foxo*Δ genotype. For all survival experiments, *n* > 85 deaths were counted per condition; *P* values are from either log-rank tests between groups (*C* and *D*) or Cox proportional hazards testing (*E*).

To assess the TFs most likely to modulate lifespan downstream of IIS in the nervous system, we first examined the endogenous expression of each TF in neurons and glia using published single-cell RNA-seq data from adult *Drosophila* brains (33). We observed that *foxo* messenger RNA (mRNA) was most highly expressed in cells expressing the canonical glial TF *repo* (Fig. 1*A*), whereas *fkh* mRNA was most highly expressed in cells expressing *neuronal Synaptobrevin* (*nSyb*), a canonical neuronally expressed gene (Fig. 2*A*). In each case, we confirmed by immunofluorescence imaging that glial localization of FOXO and neuronal localization of FKH matched the transcriptomic data, using either a green fluorescent protein (GFP)-labeled FOXO protein under the control of its native promoter (Fig. 1*B*) or antibody labeling for FKH (Fig. 2*B*). These data prompted us to hypothesize that FKH might have a greater capacity than FOXO in neurons to extend healthy lifespan.

To directly test this hypothesis, we first tested whether *foxo* overexpression in neurons or glia would extend healthy lifespan. We restricted overexpression of *foxo* to adulthood by using the GeneSwitch inducible expression system, which drives expression of UAS transgenes only when flies are fed the inducing drug RU-486 (34). Adult-onset overexpression of *foxo* using the pan-neuronal *elav-GS* driver caused a marked shortening of lifespan (Fig. 1*C*; median lifespan −18.2% and *P* = 0.0051 versus uninduced control), consistent with previous reports (17, 20). However, adult-onset overexpression of *foxo* using the pan-glial *Glia-GS* driver significantly increased healthy lifespan (Fig. 1*D*; median lifespan +8.8% and *P* = 2.0 × 10^−6^), a result that was reproducible in independent experiments (*SI Appendix*, Fig. S1*A*) and not observed for flies expressing the *Glia-GS* driver alone (*SI Appendix*, Fig. S1*B*). In addition, and consistent with previous studies examining the effects of reduced IIS in glia (31), we observed no change in fecundity for flies overexpressing FOXO in glia (*SI Appendix*, Fig. S1*C*).

Next, we assessed adult-onset neuronal overexpression of *fkh* using *elav-GS*, which led to a significant increase in lifespan (Fig. 2*C*; median lifespan +6.6% and *P* = 2.3 × 10^−7^ versus uninduced control), a result that was similarly reproducible in independent experiments (*SI Appendix*, Fig. S2*A*) and not due to the *elav-GS* driver alone (*SI Appendix*, Fig. S2*B*). Here again, we observed no change in fecundity with neuronal FKH overexpression (*SI Appendix*, Fig. S2*C*). We also tested the effects of glial adult-onset overexpression of *fkh* using *Glia-GS*; however, we observed no significant change in lifespan for these flies (Fig. 2*D*; *P* = 0.40). Taken together, these data indicate that overexpression of either *foxo* in glia or *fkh* in neurons, cell types in which these TFs are endogenously most highly expressed, can extend healthy lifespan in ways that TFs not normally expressed cannot.

### Neuronal FKH Does Not Interact with FOXO in Modulating Healthy Lifespan.

Because of the established prolongevity and antiaging effects of reduced IIS in neurons (28–30, 32) and the reported roles played by FOXA TFs in adult mammalian dopaminergic neurons (35), we decided to pursue the role of FKH in neurons more extensively. We first asked whether neuronal *fkh* overexpression would interact with other established prolongevity pathways. Previous studies have shown that ubiquitous IIS inhibition requires both FOXO and FKH to extend lifespan (12, 21). However, other studies have shown that *foxo* overexpression from the fat body and intestinal tissues does not require *foxo* expression in any other tissue to extend lifespan (36). We therefore tested whether neuronal FKH would require *foxo* expression in other tissues to extend lifespan. We found that, even in a *foxo*-null genetic background, neuronal *fkh* overexpression extended lifespan to a similar extent in both wild-type and *foxo*Δ flies (Fig. 2*E*; wild-type median lifespan +10.4% and *P* = 4.5 × 10^−5^, *foxo*Δ median lifespan +8.0% and *P* = 0.0029). Cox proportional hazards analysis confirmed that lifespan extension from increased neuronal FKH expression was independent of FOXO (*P* = 0.61), suggesting that, like *foxo* overexpression in the fat body (36), *fkh* overexpression in neurons does not require FOXO-driven transcriptional changes in other cell types to extend healthy lifespan.

Rapamycin is a well-characterized mTOR inhibitor with established prolongevity effects. Unlike the lifespan extension derived from ubiquitous IIS inhibition, the prolongevity effects of rapamycin do not require FOXO (37); in contrast, rapamycin’s effects on lifespan require FKH presence in the gut (21). To assess whether *fkh* in neurons would also be required for rapamycin’s effects, we combined RNA interference (RNAi) knockdown of *fkh* in adult neurons using *elav-GS* with rapamycin feeding starting from 2 d of age. We first found that neuronal *fkh* knockdown alone did not significantly alter lifespan (*SI Appendix*, Fig. S2*D*; *P* = 0.86). However, in contrast to the findings from *fkh* knockdown in the gut, rapamycin treatment extended lifespan to the same extent with or without neuronal *fkh* knockdown (uninduced median lifespan +11.6% and *P* = 9.0 × 10^−14^, *fkh* knockdown median lifespan +11.3% and *P* = 5.7 × 10^−12^), with no significant interaction between neuronal *fkh* knockdown and rapamycin treatment (Cox proportional hazards interaction, *P* = 0.32). Taken together, these results confirm that increased neuronal FKH is sufficient to extend lifespan in a FOXO-independent manner. They also provide preliminary support for potential differences between neuronal and intestinal FKH function with respect to pharmacological mTOR inhibition, although additional studies, for example quantifying additive effects of neuronal FKH and pharmacological mTOR inhibition, will be necessary to fully define this interaction.

### Overexpression of FKH in Neurons Improves Locomotor Function and Reduces Protein Aggregation in Aβ-Expressing Flies.

Previous studies have found that chronically reduced IIS can have beneficial effects on neuronal function during healthy aging and in disease models: in *Drosophila*, neuron-specific IIS inhibition preserves youthful neurotransmission during healthy aging (32); in *Caenorhabditis elegans*, ubiquitous IIS inhibition slows locomotor decline in the presence of the human Alzheimer’s disease Aβ peptide (38); and in mice, ubiquitous IIS inhibition preserves spatial memory behavior when Aβ is overexpressed in neurons (39). To determine whether increased *fkh* expression could produce similar protective effects against Aβ in neurons, we turned to an established *Drosophila* model of neuronal expression of Aβ_Arc_, a highly toxic and oligomer-prone form of Aβ derived from a familial form of Alzheimer’s disease [*APP*^*E693G*^ (40)]. Adult-onset expression of Aβ_Arc_ in neurons produces marked phenotypes of shortened lifespan and reduced neuromuscular function as assessed by the negative geotaxis (climbing) assay (41).

**Fig. 3.**
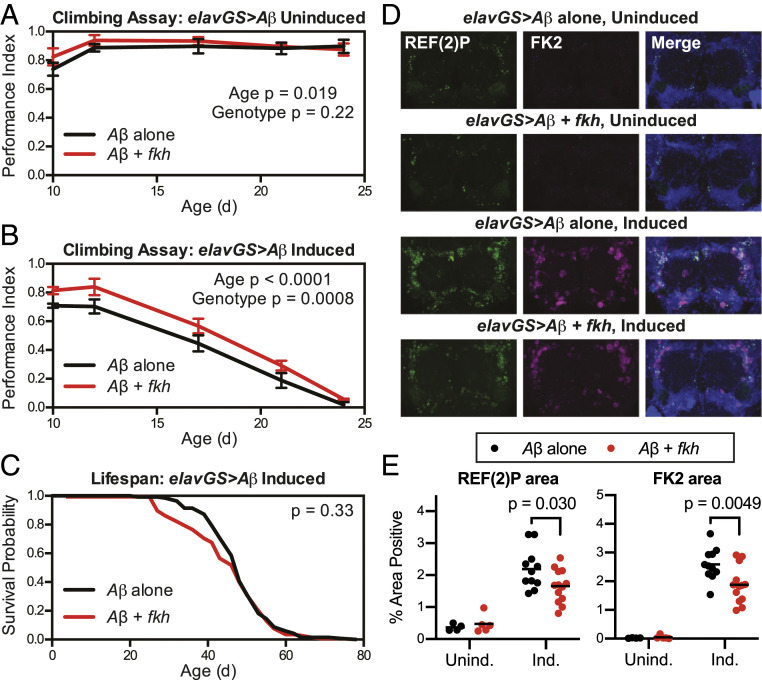
Overexpression of *fkh* in neurons improves neuromuscular function and reduces protein aggregation in the presence of Aβ. (*A* and *B*) Climbing assay results show (*A*) unaffected neuromuscular function in uninduced (vehicle control) *w*^*1118*^*;UAS-A*β_*Arc*_*/UAS-fkh;elav-GS/+* flies compared with uninduced *w*^*1118*^*;UAS-A*β_*Arc*_*/+;elav-GS/+* flies and (*B*) improved function in induced (200 μM RU-486) *w*^*1118*^*;UAS-A*β_*Arc*_*/UAS-fkh;elav-GS/+* flies compared with induced *w*^*1118*^*;UAS-A*β_*Arc*_*/+;elav-GS/+* flies. (*C*) Survival curves show no change in lifespan for induced (200 μM RU-486) *w*^*1118*^*;UAS-A*β_*Arc*_*/UAS-fkh;elav-GS/+* flies compared with induced *w*^*1118*^*;UAS-A*β_*Arc*_*/+;elav-GS/+* flies. (*D* and *E*) Immunostaining images (*D*) and quantification (*E*) show reduced accumulation of REF(2)P and polyubiquitinated protein (FK2) aggregates in induced *w*^*1118*^*;UAS-A*β_*Arc*_*/UAS-fkh;elav-GS/+* flies compared with induced *w*^*1118*^*;UAS-A*β_*Arc*_*/+;elav-GS/+* flies at 28 d of age. For climbing experiments, *n* = 3 vials of 15 flies per vial per condition; data are displayed as mean ± SEM; for survival experiments, *n* > 135 deaths were counted per condition; and for immunofluorescence experiments, *n* = 4 or 5 brains for uninduced and 11 to 13 brains for induced conditions. *P* values are from two-way ANOVA (*A* and *B*), log-rank test (*C*), or Bonferroni multiple comparisons (*E*).

We first tested whether neuronal *fkh* overexpression would protect against neuromuscular decline caused by Aβ. In the absence of RU-486, we observed no difference in climbing ability between flies with or without the *UAS-fkh* transgene (Fig. 3*A*; genotype effect *P* = 0.22 by two-way ANOVA). However, in the presence of RU-486, we found that flies overexpressing *fkh* in neurons were significantly protected from the decline in climbing activity produced by Aβ (Fig. 3*B*; genotype effect *P* = 0.0008 by two-way ANOVA), a result that was reproducible in independent experiments (*SI Appendix*, Fig. S3). At the same time, we observed that neuronal *fkh* overexpression did not produce any improvement in the shortened lifespan caused by Aβ (Fig. 3*C*; *P* = 0.33 by log-rank test). Notably, these findings are in contrast to previous findings that dietary restriction increases lifespan of the same *Drosophila* Aβ model without producing any improvement in climbing ability (42), revealing additional distinctions between the prolongevity effects of neuronal *fkh* overexpression and dietary treatments that impact nutrient-sensing pathways in other tissues.

In addition to behavioral impairments, *Drosophila* models of neurodegenerative disease are often characterized by the presence of polyubiquitinated protein aggregates and accumulation of the *Drosophila* p62/SQSTM1 ortholog REF(2)P (43). To determine whether improved proteostasis may be one mechanism by which FKH improves function in Aβ-expressing brains, we quantified the levels of REF(2)P and polyubiquitinated (FK2 antibody-positive) protein aggregates in fly brains expressing either Aβ alone or Aβ with *fkh* (Fig. 3*D*). We observed a significant reduction in the accumulation of both markers with *fkh* coexpression (Fig. 3*E*), suggesting that FKH can ameliorate the effects of Aβ on both behavioral decline and protein aggregation in the brain.

### FKH-Dependent Transcriptional Responses to Reduced IIS.

To identify potential beneficial pathways downstream of FKH in neurons, we next turned to a transcriptomic approach. Our previous studies have shown that FKH function is necessary for reduced IIS to extend *Drosophila* lifespan, using ubiquitous RNAi-mediated *fkh* knockdown in combination with overexpression of a dominant-negative kinase-dead form of the *Drosophila* insulin receptor (InR^K1409A^, or InR^DN^) (21). These studies identified an essential role of *fkh* in the gut and used RNA-seq from gut tissue to identify FKH-dependent transcriptional changes in response to reduced IIS. Given our similar results described above for neuronal *fkh* overexpression, we employed the same strategy from head tissues to identify FKH-dependent transcriptional changes in the nervous system in response to reduced IIS.

**Fig. 4.**
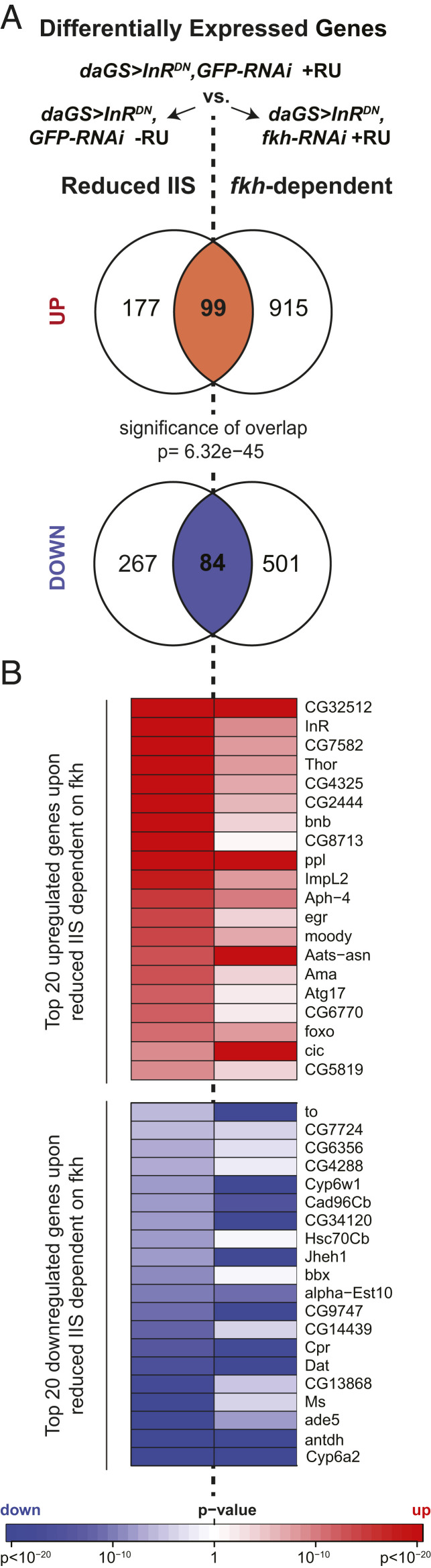
RNA-seq from head mRNA reveals *fkh*-dependent transcriptional responses to reduced IIS. (*A*) In head mRNA from flies treated with 200 μM RU-486 or vehicle control from 2 to 5 d of age, the overlap between the transcriptional response from reduced IIS compared with controls (*daGS>UAS-InR*^*DN*^*,UAS-GFP-RNAi* induced versus *daGS>UAS-InR*^*DN*^*,UAS-GFP-RNAi* uninduced) and compared with flies with reduced IIS and *fkh* knockdown (*daGS>UAS-InR*^*DN*^*,UAS-GFP-RNAi* induced versus *daGS>UAS-InR*^*DN*^*,UAS-fkh-RNAi* induced), revealed 99 shared up-regulated and 84 shared down-regulated genes, which represented a significant overlap between comparisons (*P* = 6.3 × 10^−45^ by Fisher’s exact test). (*B*) Heatmap shows *P* values for the 20 genes most significantly up-regulated or down-regulated among the shared genes between comparisons.

We isolated heads from flies with ubiquitous reduction of IIS (using the ubiquitous *da-GS* driver and *UAS-InR*^*DN*^) with or without RNAi knockdown of *fkh*. While *Drosophila* head tissues contain multiple cell types and some tissues from outside of the nervous system, we reasoned that this approach would allow us to see both neuronal and nonneuronal effects of FKH in head tissues; moreover, roughly 90% of *Drosophila* brain cells are neurons (44), so we predicted that our results would be enriched for neuronal gene expression. To help correct for off-target effects from ubiquitous activation of the RNAi machinery (45), we used a *UAS-GFP-RNAi* transgene as the control condition to compare with expression of *UAS-fkh-RNAi*. We first identified the set of genes differentially expressed in wild-type and reduced-IIS head extracts (*da-GS>UAS-InR*^*DN*^*,UAS-GFP-RNAi* with RU-486 versus *da-GS>UAS-InR*^*DN*^*,UAS-GFP-RNAi* without RU-486) and the set of genes differentially expressed in reduced-IIS heads with or without RNAi knockdown of *fkh* (*daGS>UAS-InR*^*DN*^*,UAS-GFP-RNAi* with RU-486 versus *daGS>UAS-InR*^*DN*^*,UAS-fkh-RNAi* with RU-486). When looking at the genes that overlapped between these two comparisons, we found 99 overlapping up-regulated genes and 84 overlapping down-regulated genes, which represented a highly significant overlap between comparisons (Fig. 4*A*; *P* = 6.32 × 10^−45^). As previously observed for gut tissue, these data suggest that a significant proportion of the transcriptional response to reduced IIS in the nervous system is mediated by FKH.

We next hypothesized that the most promising beneficial transcriptional responses regulated by FKH in response to reduced IIS should be indicated by genes whose expression levels revert toward wild-type levels upon *fkh* knockdown in reduced-IIS flies. We therefore looked at the most significantly up-regulated and down-regulated genes upon reduced IIS whose transcriptional changes depended on FKH expression (Fig. 4*B* and *SI Appendix*, Table S1). Among the up-regulated genes, we identified a number of genes involved in IIS whose expression reverted toward control levels with *fkh* knockdown, including *ImpL2*, *Thor*, and *foxo*, potentially indicative of feedback loops downstream of IIS that depend on FKH activity. Notably, these genes and/or their orthologs in other species have all previously been shown to modulate healthy lifespan (24, 46, 47). In addition, we noted that expression of *Atg17* was markedly increased upon reduced IIS and significantly reverted toward control levels with *fkh* knockdown (Fig. 4*B*). *Drosophila Atg17* encodes a subunit of the ATG1 kinase complex that initiates autophagosome formation to regulate the rate of autophagy (48), one of the central cellular processes that maintains proteostasis and organelle recycling in nervous system health and neurodegenerative diseases (49). We therefore decided to explore further the role of neuronal *Atg17* in *fkh*-mediated modulation of longevity.

### *Atg17* Is a Neuronal FKH Target That Can Extend Healthy Lifespan.

**Fig. 5.**
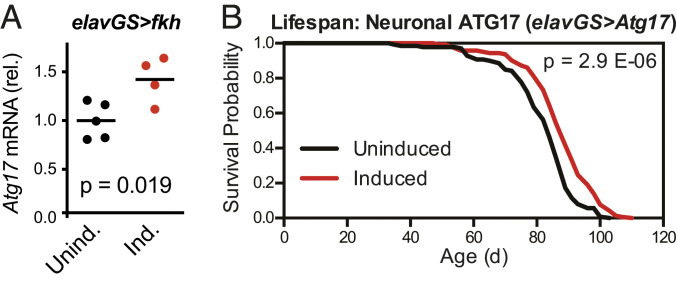
Overexpression of *fkh* in neurons increases *Atg17* expression, and overexpression of *Atg17* in neurons extends lifespan. (*A*) qPCR from head RNA shows increased mRNA levels for *Atg17* in *w*^*Dah*^*;UAS-fkh/+;elav-GS/+* flies given food containing 200 μM RU-486 from 2 to 5 d of age. *n* = 4 or 5 biological replicates of 30 heads per replicate for each condition; the *P* value is by unpaired *t* test. (*B*) Survival curves show extended lifespan for *w*^*Dah*^*;UAS-Atg17/+;elav-GS/+* flies treated with 200 μM RU-486 from 2 d of age. *n* > 135 deaths were counted per condition; the *P* value is by log-rank test.

To determine whether increased neuronal *Atg17* expression could be an important beneficial downstream effect of neuronal *fkh*, we first tested whether *Atg17* levels were increased in long-lived flies overexpressing neuronal *fkh*. qPCR analysis on heads isolated from *elavGS>UAS-fkh* flies showed a significant ∼42% increase in *Atg17* expression levels (Fig. 5*A*), consistent with the FKH-dependent modulation of *Atg17* expression we had observed in our RNA-seq results (Fig. 4*B*).

We next directly tested whether increased neuronal *Atg17* expression would be sufficient to extend healthy lifespan on its own. We overexpressed *Atg17* in adult neurons and observed a significant increase in lifespan (Fig. 5*B*; median lifespan +5.4% and *P* = 2.9 × 10^−6^). Importantly, this lifespan extension was reproducible in independent experiments (*SI Appendix*, Fig. S4*A*). These results were consistent with previous studies showing that up-regulation of other genes in autophagy-related pathways, including *Atg1*, can have prolongevity effects (50). To confirm these published studies, we overexpressed *Atg1* in adult neurons using two independent *UAS-Atg1* transgenes; in both cases, we observed some early mortality but overall significant increases in both median (+3.1 and +9.8%) and maximum (+4.2 and +12.7%) lifespan (*SI Appendix*, Fig. S4 *B* and *C*).

Finally, we assessed whether FKH overexpression in neurons would affect markers for autophagy and proteostasis in young and aged flies. We first quantified the *Drosophila* LC3 ortholog ATG8, in both its unlipidated (ATG8-I) and autophagy-associated lipidated (ATG8-II) forms (*SI Appendix*, Fig. S5 *A*–*D*); however, we did not observe any significant differences in healthy young flies and instead observed a decrease in ATG8-II levels in aged flies. However, the interpretation of LC3/ATG8 levels can be complex, as sustained increases in autophagy can reduce overall LC3/ATG8 levels in both *Becn1*^*F121A/F121A*^ mice (51) and *chico*-null flies (52). Moreover, LC3-II/ATG8-II can be difficult to interpret in nervous system cell types, where increases from its low basal levels can indicate either increased autophagy or blocked autophagic flux (53). We therefore quantified the levels of insoluble polyubiquitinated proteins as a secondary readout of proteostasis (*SI Appendix*, Fig. S5 *E*–*H*); here, we observed no differences in either young or aged *fkh*-overexpressing flies. Notably, this was in contrast to our results from Aβ-expressing flies (Fig. 3 *D* and *E*), suggesting that FKH may exert its beneficial effects on proteostasis most measurably under conditions of stress or disease. Taken together, these results suggest that increased *Atg17* expression is one, but likely not the only, beneficial effect of neuronal *fkh* overexpression, leaving open the possibility for multiple other pathways that can increase healthy lifespan downstream of neuronal FKH.

## Discussion

Here we have shown that distinct Forkhead family TFs in the *Drosophila* nervous system can modulate healthy lifespan through their activity in different cell types. We have found that FKH, rather than FOXO, can extend healthy lifespan in neurons, whereas FOXO appears to be the more relevant Forkhead family TF for glial cells. We have also demonstrated that increased FKH activity in neurons can protect against some of the detrimental effects of Aβ in *Drosophila* neurons, suggesting that FKH can play roles not only in healthy lifespan but also in pathways that modulate toxicity in neurodegenerative diseases. Finally, we have identified *Atg17* as one lifespan-modulating gene downstream of FKH in neurons in response to reduced IIS, although other pathways downstream of FKH are very likely to play additional beneficial roles.

These findings help shed light on an apparent paradox from previous studies on the modulation of lifespan by neurons: While decreased IIS in neurons extends healthy lifespan (29), the canonical downstream effect of increased FOXO activity in neurons shortens lifespan (17, 20). Based on single-cell transcriptomic data (33) and our immunostaining results from the adult *Drosophila* brain, we now suggest that the endogenous expression patterns of multiple TFs among different cell types can help inform the interpretation of these results. Specifically, our results place neurons alongside differentiated intestinal cells (21) as cell types in which FKH, rather than FOXO, is sufficient to mediate prolongevity effects. Notably, these results are in agreement with other recent studies pointing to the limited expression of FOXO in *Drosophila* neurons compared with other cell types (54). Our results also place glial cells alongside the fat body (24) and some gut cell populations (20) as *Drosophila* cell types in which FOXO can mediate similar effects. Our results also complement our recent findings that glia-specific IIS inhibition can extend healthy lifespan in a FOXO-dependent manner (31). Finally, our results do not exclude the possibility that other TFs downstream of IIS in the nervous system can modulate longevity; indeed, recent studies have shown that knockdown of the ETS family TF *Eip74EF* in neurons can extend healthy lifespan (20), and other TFs are likely to play additional roles in nervous system aging.

Downstream of FKH, our transcriptomic results suggest that, as was previously observed for gut tissues (21), a significant proportion of the downstream effects of reduced IIS in the nervous system are FKH-dependent. However, whereas intestinal FKH achieves some of its protective effects through up-regulation of nutrient transporters (21), we find here that one of the strongest FKH-dependent changes in the nervous system is in the transcription of *Atg17*. Taken together with published chromatin immunoprecipitation-sequencing studies identifying the *Atg17* genomic region as one bound by both FKH (55) and FOXO (56), our results suggest that *Atg17* could be a direct target gene of FKH in neurons. Our further experiments show here that increased expression of *Atg17* in neurons is sufficient on its own to extend lifespan. As the primary known function of ATG17 is in the ATG1 kinase complex that initiates autophagy (48), our results suggest that autophagy could be one beneficial effect downstream of neuronal FKH, consistent with previous studies and our own findings here showing that increased neuronal expression of *Atg1* (50) or *Atg8* (57) can extend healthy lifespan in *Drosophila*. In addition, increased neuronal autophagy could be one pathway explaining how increased neuronal *fkh* expression can mitigate some of the detrimental effects of Aβ in neurons. Here too, our results are consistent with previous studies showing beneficial effects of either genetic (58, 59) or pharmacological (60) up-regulation of autophagy in animal models of Aβ toxicity. However, the lack of marked changes in insoluble ubiquitinated protein accumulation in *fkh*-overexpressing flies during healthy aging (*SI Appendix*, Fig. S5) suggests either that autophagy is not dramatically changed with neuronal *fkh* overexpression in healthy aging or that more specific tools such as fluorescently labeled endogenous ATG8A (61) may be needed to adequately assess autophagy in aging.

Importantly, our results also open the door to other factors in addition to autophagy that could contribute to the beneficial effects of FKH on healthy lifespan. Our transcriptomic results reveal a large number of genes whose response to lower IIS is dependent on FKH, and many of these genes may contribute to or be indicative of beneficial pathways downstream of FKH. For instance, *Cyp6a2*, *Cyp6w1*, and *Hsc70Cb* are involved in stress responses involving cytochrome P450-mediated xenobiotic detoxification and chaperone-mediated protein folding; their FKH-dependent transcriptional regulation could indicate that these would be fruitful pathways to pursue in future research on neuron-mediated modulation of longevity. Other pathways beyond these genes could also be promising areas for study: For example, both FOXO and FKH have been identified as mediators of immune responses in *Drosophila* (21, 62–64). We propose that deciphering the specific cell types in which either TF might be acting in the immune response could therefore be key to uncovering additional prolongevity factors regulated by FKH and FOXO.

Our findings have implications for research in other animal species, where it will be important to consider whether the pattern of TF expression among cell types is conserved for orthologous TFs. For example, mammalian genomes contain multiple genes encoding both FOXA and FOXO family members, each with distinct effects on age-related phenotypes (reviewed in ref. 65) that may result from their cell type-specific expression patterns. Importantly, transcriptomic data from sorted neurons and glial cell populations from both adult mouse (66) and adult human (67) brain tissues show gene expression patterns that only partially match the patterns observed in *Drosophila*. These data show low expression of FOXA family members in all mouse and human brain cell types, with distinct expression patterns of FOXO family members in different cell types including some neuronal populations. These patterns are largely consistent with previously observed effects of FOXO3 on autophagy in mouse striatal neurons (68) and for FOXO6 in modulating memory consolidation in mouse hippocampal neurons (69). However, high-throughput datasets can miss vital genes expressed at low levels or in small subpopulations of cells: For example, FOXA family members are expressed in adult mouse midbrain dopaminergic neurons, where they play an important role in the regulation of genes encoding essential enzymes for dopamine biosynthesis (35). When considered in the context of our current results, the cell type-specific expression patterns of TFs, as determined by both high-throughput and targeted studies, will be useful guides for future studies on aging and longevity.

Finally, our results underscore the need for future studies to consider cell-type specificity of TF action in order to understand physiological processes that involve multiple heterogeneous tissues. In this regard, studies can draw on the growing knowledge of cell type-specific TF action from established fields such as immunology and endocrinology. For instance, recent studies have identified the distinct TFs acting in regulatory T cells and T helper 17 cells to mediate either antiinflammatory or proinflammatory transcriptional responses to transforming growth factor β stimulation, respectively (70, 71). Similarly, estrogen signaling achieves a plethora of outcomes in organ systems including cardiovascular, musculoskeletal, and reproductive systems, in each case via cell type-specific consequences of distinct TFs acting in concert with the estrogen receptor (72, 73). Our results now add neuronal FKH and glial FOXO activity downstream of IIS as an additional example of how cell type-specific responses to similar stimuli are maintained by distinct TF expression patterns.

In summary, we have identified neuronal FKH and glial FOXO as cell type-specific targets to extend healthy lifespan in *Drosophila*. Our results match the basal expression patterns for these TFs, underscoring the need to consider where TFs are normally active before using interventions that attempt to modulate their activity. Cell type-specific effects will therefore be an essential consideration for understanding and addressing the complexities of aging, not only in the *Drosophila* nervous system but also in other heterogeneous tissues and ultimately in other species including humans.

## Materials and Methods

### Fly Stocks and Husbandry.

*Drosophila* stocks were maintained and experiments were conducted at 25 °C on a 12-h:12-h light:dark cycle at 65% humidity, on food containing 10% (weight/volume; wt/vol) brewer’s yeast, 5% (wt/vol) sucrose, and 1.5% (wt/vol) agar. The wild-type stock *Dahomey* was collected in 1970 in Dahomey (now Benin) and has since been maintained in large population cages with overlapping generations on a 12-h:12-h light:dark cycle at 25 °C. The *white Dahomey* (*w*^*Dah*^) stock was derived by incorporation of the *w*^*1118*^ allele into the outbred *Dahomey* background by backcrossing. All fly stocks in this study were backcrossed for six or more generations into the outbred *w*^*Dah*^ background (Figs. 1, 2, 4, and 5) or an inbred *w*^*1118*^ background (Fig. 3). Additional information on stocks used and media preparation is available in *SI Appendix*.

### Survival Analysis.

Lifespan assays were carried out as described in detail in ref. 74. Female flies were used for all experiments. From the eggs collected for each set of parental crosses, the progeny that emerged as adults within a 24-h window were collected and allowed to mate for 48 h, after which they were separated into single-sex vials containing either drug- or vehicle-containing food at a density of 15 individuals per vial. Vials were kept either individually or in DrosoFlippers (drosoflipper.com) for ease of regular transfer to fresh vials. Flies were transferred to fresh vials three times per week, with deaths and censors scored during each transfer. Microsoft Excel (template described in ref. 74) was used to calculate survival proportions.

### Additional Methods.

Methods for other techniques (climbing, fecundity measurements, immunofluorescence, RNA-seq, qPCR, and Western blots) are available in *SI Appendix*.

### Statistical Analysis.

The statistical test used for each experiment is indicated in the figure legends. Log-rank tests were performed in Microsoft Excel (template described in ref. 74), and Cox proportional hazards tests were performed in R using the survival package. ANOVA or *t* test analyses were performed in GraphPad Prism 8.4. For all statistical tests, *P* < 0.05 was considered significant.

## Supplementary Material

Supplementary File

## Data Availability

The RNA-seq data analyzed in this article are freely available at ArrayExpress (https://www.ebi.ac.uk/arrayexpress/) (accession no. E-MTAB-9017) (75).

## References

[1] M. C. Petersen, G. I. Shulman, Mechanisms of insulin action and insulin resistance. Physiol. Rev. 98, 2133–2223 (2018). 30067154 10.1152/physrev.00063.2017 PMC6170977

[2] N. Alic, L. Partridge, Death and dessert: Nutrient signalling pathways and ageing. Curr. Opin. Cell Biol. 23, 738–743 (2011). 21835601 10.1016/j.ceb.2011.07.006 PMC4335171

[3] L. Fontana, L. Partridge, V. D. Longo, Extending healthy life span—From yeast to humans. Science 328, 321–326 (2010). 20395504 10.1126/science.1172539 PMC3607354

[4] D. Gems, L. Partridge, Genetics of longevity in model organisms: Debates and paradigm shifts. Annu. Rev. Physiol. 75, 621–644 (2013). 23190075 10.1146/annurev-physiol-030212-183712

[5] S. J. Broughton., Longer lifespan, altered metabolism, and stress resistance in *Drosophila* from ablation of cells making insulin-like ligands. Proc. Natl. Acad. Sci. U.S.A. 102, 3105–3110 (2005). 15708981 10.1073/pnas.0405775102 PMC549445

[6] S. Grönke, D. F. Clarke, S. Broughton, T. D. Andrews, L. Partridge, Molecular evolution and functional characterization of *Drosophila* insulin-like peptides. PLoS Genet. 6, e1000857 (2010). 20195512 10.1371/journal.pgen.1000857 PMC2829060

[7] D. J. Clancy., Extension of life-span by loss of CHICO, a *Drosophila* insulin receptor substrate protein. Science 292, 104–106 (2001). 11292874 10.1126/science.1057991

[8] M. Tatar., A mutant *Drosophila* insulin receptor homolog that extends life-span and impairs neuroendocrine function. Science 292, 107–110 (2001). 11292875 10.1126/science.1057987

[9] C. Slack., Regulation of lifespan, metabolism, and stress responses by the *Drosophila* SH2B protein, Lnk. PLoS Genet. 6, e1000881 (2010). 20333234 10.1371/journal.pgen.1000881 PMC2841611

[10] M. Holzenberger., IGF-1 receptor regulates lifespan and resistance to oxidative stress in mice. Nature 421, 182–187 (2003). 12483226 10.1038/nature01298

[11] C. Selman., Evidence for lifespan extension and delayed age-related biomarkers in insulin receptor substrate 1 null mice. FASEB J. 22, 807–818 (2008). 17928362 10.1096/fj.07-9261com

[12] C. Slack, M. E. Giannakou, A. Foley, M. Goss, L. Partridge, dFOXO-independent effects of reduced insulin-like signaling in *Drosophila*. Aging Cell 10, 735–748 (2011). 21443682 10.1111/j.1474-9726.2011.00707.x PMC3193374

[13] C. Slack., The Ras-Erk-ETS-signaling pathway is a drug target for longevity. Cell 162, 72–83 (2015). 26119340 10.1016/j.cell.2015.06.023 PMC4518474

[14] Y. Suh., Functionally significant insulin-like growth factor I receptor mutations in centenarians. Proc. Natl. Acad. Sci. U.S.A. 105, 3438–3442 (2008). 18316725 10.1073/pnas.0705467105 PMC2265137

[15] V. Kontis., Future life expectancy in 35 industrialised countries: Projections with a Bayesian model ensemble. Lancet 389, 1323–1335 (2017). 28236464 10.1016/S0140-6736(16)32381-9 PMC5387671

[16] C. Kenyon, J. Chang, E. Gensch, A. Rudner, R. Tabtiang, A *C. elegans* mutant that lives twice as long as wild type. Nature 366, 461–464 (1993). 8247153 10.1038/366461a0

[17] D. S. Hwangbo, B. Gershman, M. P. Tu, M. Palmer, M. Tatar, *Drosophila* dFOXO controls lifespan and regulates insulin signalling in brain and fat body. Nature 429, 562–566 (2004). 15175753 10.1038/nature02549

[18] L. Broer., GWAS of longevity in CHARGE consortium confirms APOE and FOXO3 candidacy. J. Gerontol. A Biol. Sci. Med. Sci. 70, 110–118 (2015). 25199915 10.1093/gerona/glu166 PMC4296168

[19] J. Deelen., A meta-analysis of genome-wide association studies identifies multiple longevity genes. Nat. Commun. 10, 3669 (2019). 31413261 10.1038/s41467-019-11558-2 PMC6694136

[20] A. J. Dobson., Longevity is determined by ETS transcription factors in multiple tissues and diverse species. PLoS Genet. 15, e1008212 (2019). 31356597 10.1371/journal.pgen.1008212 PMC6662994

[21] E. Bolukbasi., Intestinal Fork Head regulates nutrient absorption and promotes longevity. Cell Rep. 21, 641–653 (2017). 29045833 10.1016/j.celrep.2017.09.042 PMC5656751

[22] G. Martínez Corrales, N. Alic, Evolutionary conservation of transcription factors affecting longevity. Trends Genet. 36, 373–382 (2020). 32294417 10.1016/j.tig.2020.02.003

[23] F. Demontis, N. Perrimon, FOXO/4E-BP signaling in *Drosophila* muscles regulates organism-wide proteostasis during aging. Cell 143, 813–825 (2010). 21111239 10.1016/j.cell.2010.10.007 PMC3066043

[24] M. E. Giannakou., Long-lived *Drosophila* with over-expressed dFOXO in adult fat body. Science 305, 361 (2004). 15192154 10.1126/science.1098219

[25] L. S. Tain., A proteomic atlas of insulin signalling reveals tissue-specific mechanisms of longevity assurance. Mol. Syst. Biol. 13, 939 (2017). 28916541 10.15252/msb.20177663 PMC5615923

[26] T. Niccoli, L. Partridge, Ageing as a risk factor for disease. Curr. Biol. 22, R741–R752 (2012). 22975005 10.1016/j.cub.2012.07.024

[27] Office for National Statistics, “Deaths registered in England and Wales: 2014” (2015). https://www.ons.gov.uk/peoplepopulationandcommunity/birthsdeathsandmarriages/deaths/bulletins/deathsregistrationsummarytables/2018. Accessed 20 April 2020.

[28] L. Kappeler., Brain IGF-1 receptors control mammalian growth and lifespan through a neuroendocrine mechanism. PLoS Biol. 6, e254 (2008). 18959478 10.1371/journal.pbio.0060254 PMC2573928

[29] H. Augustin., Impact of insulin signaling and proteasomal activity on physiological output of a neuronal circuit in aging *Drosophila melanogaster*. Neurobiol. Aging 66, 149–157 (2018). 29579685 10.1016/j.neurobiolaging.2018.02.027 PMC5933513

[30] M. Z. B. H. Ismail., The *Drosophila* insulin receptor independently modulates lifespan and locomotor senescence. PLoS One 10, e0125312 (2015). 26020640 10.1371/journal.pone.0125312 PMC4447345

[31] N. S. Woodling, A. Rajasingam, L. J. Minkley, A. Rizzo, L. Partridge, Independent glial subtypes delay development and extend healthy lifespan upon reduced insulin-PI3K signalling. BMC Biol. 18, 124 (2020). 32928209 10.1186/s12915-020-00854-9 PMC7490873

[32] H. Augustin., Reduced insulin signaling maintains electrical transmission in a neural circuit in aging flies. PLoS Biol. 15, e2001655 (2017). 28902870 10.1371/journal.pbio.2001655 PMC5597081

[33] K. Davie., A single-cell transcriptome atlas of the aging *Drosophila* brain. Cell 174, 982–998.e20 (2018). 29909982 10.1016/j.cell.2018.05.057 PMC6086935

[34] T. Osterwalder, K. S. Yoon, B. H. White, H. Keshishian, A conditional tissue-specific transgene expression system using inducible GAL4. Proc. Natl. Acad. Sci. U.S.A. 98, 12596–12601 (2001). 11675495 10.1073/pnas.221303298 PMC60099

[35] A. Pristerà., Transcription factors FOXA1 and FOXA2 maintain dopaminergic neuronal properties and control feeding behavior in adult mice. Proc. Natl. Acad. Sci. U.S.A. 112, E4929–E4938 (2015). 26283356 10.1073/pnas.1503911112 PMC4568236

[36] N. Alic., Cell-nonautonomous effects of dFOXO/DAF-16 in aging. Cell Rep. 6, 608–616 (2014). 24508462 10.1016/j.celrep.2014.01.015 PMC3969275

[37] I. Bjedov., Mechanisms of life span extension by rapamycin in the fruit fly *Drosophila melanogaster*. Cell Metab. 11, 35–46 (2010). 20074526 10.1016/j.cmet.2009.11.010 PMC2824086

[38] E. Cohen, J. Bieschke, R. M. Perciavalle, J. W. Kelly, A. Dillin, Opposing activities protect against age-onset proteotoxicity. Science 313, 1604–1610 (2006). 16902091 10.1126/science.1124646

[39] E. Cohen., Reduced IGF-1 signaling delays age-associated proteotoxicity in mice. Cell 139, 1157–1169 (2009). 20005808 10.1016/j.cell.2009.11.014 PMC3017511

[40] D. C. Crowther., Intraneuronal Abeta, non-amyloid aggregates and neurodegeneration in a *Drosophila* model of Alzheimer’s disease. Neuroscience 132, 123–135 (2005). 15780472 10.1016/j.neuroscience.2004.12.025

[41] O. Sofola., Inhibition of GSK-3 ameliorates Abeta pathology in an adult-onset *Drosophila* model of Alzheimer’s disease. PLoS Genet. 6, e1001087 (2010). 20824130 10.1371/journal.pgen.1001087 PMC2932684

[42] F. Kerr., Dietary restriction delays aging, but not neuronal dysfunction, in *Drosophila* models of Alzheimer’s disease. Neurobiol. Aging 32, 1977–1989 (2011). 19969390 10.1016/j.neurobiolaging.2009.10.015 PMC3176895

[43] K. J. Kinghorn., A *Drosophila* model of neuronopathic Gaucher disease demonstrates lysosomal-autophagic defects and altered mTOR signalling and is functionally rescued by rapamycin. J. Neurosci. 36, 11654–11670 (2016). 27852774 10.1523/JNEUROSCI.4527-15.2016 PMC5125225

[44] M. C. Kremer, C. Jung, S. Batelli, G. M. Rubin, U. Gaul, The glia of the adult *Drosophila* nervous system. Glia 65, 606–638 (2017). 28133822 10.1002/glia.23115 PMC5324652

[45] N. Alic., Detrimental effects of RNAi: A cautionary note on its use in *Drosophila* ageing studies. PLoS One 7, e45367 (2012). 23028964 10.1371/journal.pone.0045367 PMC3444450

[46] N. Alic, M. P. Hoddinott, G. Vinti, L. Partridge, Lifespan extension by increased expression of the *Drosophila* homologue of the IGFBP7 tumour suppressor. Aging Cell 10, 137–147 (2011). 21108726 10.1111/j.1474-9726.2010.00653.x PMC3042147

[47] B. M. Zid., 4E-BP extends lifespan upon dietary restriction by enhancing mitochondrial activity in *Drosophila*. Cell 139, 149–160 (2009). 19804760 10.1016/j.cell.2009.07.034 PMC2759400

[48] P. Nagy., Atg17/FIP200 localizes to perilysosomal Ref(2)P aggregates and promotes autophagy by activation of Atg1 in *Drosophila*. Autophagy 10, 453–467 (2014). 24419107 10.4161/auto.27442 PMC4077884

[49] R. A. Nixon, The role of autophagy in neurodegenerative disease. Nat. Med. 19, 983–997 (2013). 23921753 10.1038/nm.3232

[50] M. Ulgherait, A. Rana, M. Rera, J. Graniel, D. W. Walker, AMPK modulates tissue and organismal aging in a non-cell-autonomous manner. Cell Rep. 8, 1767–1780 (2014). 25199830 10.1016/j.celrep.2014.08.006 PMC4177313

[51] Á. F. Fernández., Disruption of the beclin 1-BCL2 autophagy regulatory complex promotes longevity in mice. Nature 558, 136–140 (2018). 29849149 10.1038/s41586-018-0162-7 PMC5992097

[52] I. Bjedov., Fine-tuning autophagy maximises lifespan and is associated with changes in mitochondrial gene expression in *Drosophila*. PLoS Genet. 16, e1009083 (2020). 33253201 10.1371/journal.pgen.1009083 PMC7738165

[53] D. J. Klionsky., Guidelines for the use and interpretation of assays for monitoring autophagy (3rd edition). Autophagy 12, 1–222 (2016). 26799652 10.1080/15548627.2015.1100356 PMC4835977

[54] A. R. Poe., Low FoxO expression in *Drosophila* somatosensory neurons protects dendrite growth under nutrient restriction. eLife 9, e53351 (2020). 32427101 10.7554/eLife.53351 PMC7308081

[55] Q. Lan., FoxA transcription factor Fork Head maintains the intestinal stem/progenitor cell identities in *Drosophila*. Dev. Biol. 433, 324–343 (2018). 29108672 10.1016/j.ydbio.2017.09.002

[56] A. Birnbaum, X. Wu, M. Tatar, N. Liu, H. Bai, Age-dependent changes in transcription factor FOXO targeting in female *Drosophila*. Front. Genet. 10, 312 (2019). 31134124 10.3389/fgene.2019.00312 PMC6514159

[57] A. Simonsen., Promoting basal levels of autophagy in the nervous system enhances longevity and oxidant resistance in adult *Drosophila*. Autophagy 4, 176–184 (2008). 18059160 10.4161/auto.5269

[58] F. Pickford., The autophagy-related protein beclin 1 shows reduced expression in early Alzheimer disease and regulates amyloid β accumulation in mice. J. Clin. Invest. 118, 2190–2199 (2008). 18497889 10.1172/JCI33585 PMC2391284

[59] A. Caccamo, V. De Pinto, A. Messina, C. Branca, S. Oddo, Genetic reduction of mammalian target of rapamycin ameliorates Alzheimer’s disease-like cognitive and pathological deficits by restoring hippocampal gene expression signature. J. Neurosci. 34, 7988–7998 (2014). 24899720 10.1523/JNEUROSCI.0777-14.2014 PMC4044255

[60] P. Spilman., Inhibition of mTOR by rapamycin abolishes cognitive deficits and reduces amyloid-β levels in a mouse model of Alzheimer’s disease. PLoS One 5, e9979 (2010). 20376313 10.1371/journal.pone.0009979 PMC2848616

[61] K. Hegedűs., The Ccz1-Mon1-Rab7 module and Rab5 control distinct steps of autophagy. Mol. Biol. Cell 27, 3132–3142 (2016). 27559127 10.1091/mbc.E16-03-0205 PMC5063620

[62] M. S. Dionne, L. N. Pham, M. Shirasu-Hiza, D. S. Schneider, Akt and FOXO dysregulation contribute to infection-induced wasting in *Drosophila*. Curr. Biol. 16, 1977–1985 (2006). 17055976 10.1016/j.cub.2006.08.052

[63] D. Varma, M. H. Bülow, Y. Y. Pesch, G. Loch, M. Hoch, Forkhead, a new cross regulator of metabolism and innate immunity downstream of TOR in *Drosophila*. J. Insect Physiol. 69, 80–88 (2014). 24842780 10.1016/j.jinsphys.2014.04.006

[64] C. Fink., Intestinal FoxO signaling is required to survive oral infection in *Drosophila*. Mucosal Immunol. 9, 927–936 (2016). 26627459 10.1038/mi.2015.112

[65] R. Martins, G. J. Lithgow, W. Link, Long live FOXO: Unraveling the role of FOXO proteins in aging and longevity. Aging Cell 15, 196–207 (2016). 26643314 10.1111/acel.12427 PMC4783344

[66] Y. Zhang., An RNA-sequencing transcriptome and splicing database of glia, neurons, and vascular cells of the cerebral cortex. J. Neurosci. 34, 11929–11947 (2014). 25186741 10.1523/JNEUROSCI.1860-14.2014 PMC4152602

[67] Y. Zhang., Purification and characterization of progenitor and mature human astrocytes reveals transcriptional and functional differences with mouse. Neuron 89, 37–53 (2016). 26687838 10.1016/j.neuron.2015.11.013 PMC4707064

[68] E. Pino., FOXO3 determines the accumulation of α-synuclein and controls the fate of dopaminergic neurons in the substantia nigra. Hum. Mol. Genet. 23, 1435–1452 (2014). 24158851 10.1093/hmg/ddt530

[69] D. A. M. Salih., FoxO6 regulates memory consolidation and synaptic function. Genes Dev. 26, 2780–2801 (2012). 23222102 10.1101/gad.208926.112 PMC3533081

[70] S. Tanaka., Trim33 mediates the proinflammatory function of Th17 cells. J. Exp. Med. 215, 1853–1868 (2018). 29930104 10.1084/jem.20170779 PMC6028517

[71] S. Zhang., Reversing SKI-SMAD4-mediated suppression is essential for T_H_17 cell differentiation. Nature 551, 105–109 (2017). 29072299 10.1038/nature24283 PMC5743442

[72] J. Gertz., Distinct properties of cell-type-specific and shared transcription factor binding sites. Mol. Cell 52, 25–36 (2013). 24076218 10.1016/j.molcel.2013.08.037 PMC3811135

[73] N. Heldring., Estrogen receptors: How do they signal and what are their targets. Physiol. Rev. 87, 905–931 (2007). 17615392 10.1152/physrev.00026.2006

[74] M. D. W. Piper, L. Partridge, “Protocols to study aging in *Drosophila*” in Methods in Molecular Biology, C. Dahmann, Ed. (Springer, 2016), pp. 291–302. 10.1007/978-1-4939-6371-3_18 PMC5507281 27730590

[75] D. Ivanov, E. Bolukbasi, N. Woodling, Cell-type-specific modulation of longevity by Forkhead family transcription factors in the nervous system. ArrayExpress (The European Bioinformatics Institute). https://www.ebi.ac.uk/arrayexpress/experiments/E-MTAB-9017/. Deposited 25 February 2020.

