# Supplementary material for: Cell type-specific modulation of healthspan by Forkhead family transcription factors in the nervous system
Source: Proc Natl Acad Sci U S A. 2021 Feb 15;118(8):e2011491118. doi: 10.1073/pnas.2011491118 (PMC7923679; doi:10.1073/pnas.2011491118)
Supplement: Supplementary File [file pnas.2011491118.sapp.pdf]

PNAS
www.pnas.org

Supplementary Information for

Cell type-specific modulation of healthspan by Forkhead family transcription factors in the nervous system

Ekin Bolukbasi[a,b,1], Nathaniel S. Woodling[a,b,1], Dobril K. Ivanov[c,d], Jennifer Adcott[a,b], Andrea Foley[a,b], Arjunan Rajasingam[a,b], Lauren M. Gittings[a,b], Benjamin Aleyakpo[a,b], Teresa Niccoli[a,b], Janet M. Thornton[c], and Linda Partridge[a,b,e,2]

[a] Institute of Healthy Ageing, University College London, London WC1E 6BT, United Kingdom

[b] Department of Genetics, Evolution and Environment, University College London, London WC1E 6BT, United Kingdom

[c] European Bioinformatics Institute, European Molecular Biology Laboratory, Cambridge CB10 1SD, United Kingdom

[d] UK Dementia Research Institute, Cardiff University, Cardiff CF24 4HQ, United Kingdom

[e] Department of Biological Mechanisms of Ageing, Max Planck Institute for Biology of Ageing, 50931 Cologne, Germany

[1] These authors contributed equally to this work.

[2] Corresponding Author: Linda Partridge
Email: l.partridge@ucl.ac.uk

This PDF file includes:

SI Methods
Figures S1 to S5
Table S1
SI References

SI Methods

Fly stocks. Fly stocks used in this study were *elav-GS*^{301} (Figures 1, 2, 5, S2, S4A, and S5) (1); *elav-GS*^{Tricoire} (Figures 3, S3, and S4B-C, with an *elav-GS* insertion widely used for neurodegenerative disease models; a kind gift of H. Tricoire, described in (2)); *GSG3285-1* (also called Glia-GS; a kind gift of M. Saitoe, described in (3, 4)); *da-GS* (5); *UAS-fkh-HA* (6); *UAS-foxo* (7); *foxoΔ* (8); *foxo-GFP-FLAG* (Bloomington Stock Center #38644), *UAS-A*β_{Arc} (9); *UAS-InR*^{DN} (Bloomington Stock Center #8252); *UAS-GFP-RNAi* (10); *UAS-fkh-RNAi* (Vienna Stock Center #37062); *UAS-Atg17-GFP* (a kind gift of Gábor Juhász, (11)); *UAS-Atg1*^{GS10797} (Kyoto Stock Center); and *UAS-Atg1*^{6B} (Bloomington Stock Center #51655).

Addition of RU-486 or rapamycin to fly food. For all experiments involving chemical additives to fly food, the compound was dissolved in a stock solution of ethanol and added to the fly food while it was still liquid but had cooled to 50ºC. The stock solution was added to the food, mixed well, dispensed into individual fly vials, and allowed to cool to room temperature overnight before storage at 4ºC. On the day of experiments, food vials were warmed to room temperature before being used. RU-486 (Sigma) was added to the food at a final concentration of 200µM, with equivalent amounts of ethanol used as the vehicle control condition. Rapamycin (LC Laboratories) was added to the food at a final concentration of 100µM, with equivalent amounts of ethanol used as the vehicle control condition. For all experiments, flies were allowed to develop on control food without any additives, then divided into groups to be put on vehicle- or drug-containing food 2 days after emerging as adults.

Climbing (negative geotaxis) analysis. For each genotype analyzed in climbing assays, three vials of control food and three vials of food containing RU-486 (200µM), each containing 15 flies, were maintained as in lifespan studies and tested for climbing once to twice per week as described by (12). Briefly, all flies from a vial were placed together in a vertical column (25cm long, 1.5cm diameter), tapped to the bottom of the column, and allowed to climb towards the top of the column. After 45 seconds, flies reaching the top 5cm and flies remaining at the bottom 5cm of the column were counted. Three trials were performed at 1-minute intervals for each vial. The number of flies at the top (n_{top}), the number of flies at the bottom (n_{bottom}), and the total number of flies (n_{tot}) were recorded for each trial. The performance index (PI) was defined as $PI = ½ (n_{tot} + n_{top} - n_{bottom}) / n_{tot}$, and the mean PI was then calculated among the trials for each vial on each day.

Fecundity measurements. Ten female flies per vial were allowed to lay eggs on vials containing vehicle or RU-486-containing food for less than 24 h, after which the vials were frozen to prevent larval hatching. On the day of counting, the vials were thawed, and an experimenter blinded to the genotypes and conditions counted eggs from each vial manually. From the duration of egg laying (proportion of 24h) and the number of females in each vial, a mean value of eggs per female per 24h was calculated for each vial. The data presented are the cumulative sums of these values from the three timepoints (1, 2, and 3 weeks of age) collected for each vial.

SCope database images. Images from the *SCope* database (13) were obtained from http://scope.aertslab.org/ using the following settings: *Drosophila*, Brain, Aerts-Fly-AdultBrain-Filtered-57k, 'yes' Log transform, 'yes' CPM normalize, 'yes' Expression-based plotting, and default values for all other settings.

Immunofluorescence. Adult brains were dissected in PBT (PBS with 0.5% Triton X-100), fixed in 4% PFA in PBT for 20 min at room temperature, washed in PBT four times for 20 min, blocked in PBT containing 5% Horse Serum (PBT-HS) for 1 hour, incubated in PBT-HS containing the primary antibody overnight at 4°C, washed in PBT four times for 20 min, incubated in PBT-HS containing the secondary antibody overnight at 4°C, washed four times with PBT for 20 min, and finally mounted on slides in Vectashield containing DAPI (Vector Labs). Antibodies were rabbit anti-REF(2)P (Abcam 178440, 1:200); mouse anti-polyubiquitinated proteins (FK2, Millipore 04-263, 1:200); mouse anti-REPO (Developmental Studies Hybridoma Bank 8D12-S, 1:500), rabbit anti-FKH (a kind gift of D. Andrew, (14) 1:500); secondary goat anti-mouse AlexaFluor-568 (Invitrogen A11019, 1:250), and secondary goat-anti-rabbit AlexaFluor-488 (Invitrogen A11008, 1:250).

For images of FOXO, FKH, and REPO localization, image stacks were obtained on a Zeiss LSM700 confocal microscope using a 63x objective. For quantification of REF(2)P and ubiquitin (FK2) staining, image stacks of specimens were obtained using a 20× objective for imaging of the entire central brain. Stacks of 3 μm Z-steps and 6 images per stack were taken to capture the full depth of the cell body layers dorsal, ventral, and lateral to the antennal lobes. Images were quantified by an experimenter blinded to genotype using ImageJ software. Briefly, confocal stacks were merged into a single plane, then central brain region of each brain was manually traced using the DAPI channel. Thresholds were then set for REF(2)P and ubiquitin (with the same threshold used for all images), and the area above threshold within the region of interest measured.

RNA-Seq sample and library preparation. Total RNA from fly heads (30 heads per sample) was extracted using the Qiagen total RNA isolation kit and quantified on an Agilent 2100 Bioanalyzer. Sample concentration and purity of RNA was measured on a NanoDrop spectrophotometer, and RNA integrity was assessed on an Agilent 2100 Bioanalyzer. Samples were processed using Illumina's TruSeq Stranded mRNA LT sample preparation kit (p/n RS-122-2101) according to manufacturer's instructions. Deviations from the protocol were as follows: 250ng total RNA was used as starting material; fragmentation was carried out for 10 minutes; and 14 cycles of PCR were used.

Briefly, mRNA was isolated from total RNA using Oligo dT beads to pull down poly-adenylated transcripts. The purified mRNA was fragmented using chemical fragmentation (heat and divalent metal cation) and primed with random hexamers. Strand-specific first strand cDNA was generated using Reverse Transcriptase and Actinomycin D to allow for RNA-dependent synthesis while preventing spurious DNA-dependent synthesis. The second cDNA strand was synthesized using dUTP in place of dTTP, to mark the second strand.

The resultant cDNA was then "A-tailed" at the 3' end to prevent self-ligation and adapter dimerization. Full length TruSeq adaptors, containing a T overhang, were ligated to the A-Tailed cDNA. These adaptors contained sequences that allow the libraries to be uniquely identified by way of a 6bp Index sequence. Successfully ligated fragments were enriched with 14 cycles of PCR. The polymerase used was unable to read through uracil, so only the first strand was amplified, thus making the library strand-specific.

Sequencing. Libraries to be multiplexed in the same run were pooled in equimolar quantities, calculated from Qubit and Bioanalyzer fragment analysis. Samples were sequenced on the NextSeq 500 instrument (Illumina, San Diego, US) using a 43bp paired end run resulting in >15million reads per sample. Sequencing was carried out by UCL Genomics at the UCL GOS Institute of Child Health.

Alignment and differential expression of RNA-Seq data. Quality control of the raw fastq files was performed using FastQC v0.11.4, http://www.bioinformatics.babraham.ac.uk/projects/fastqc/. The raw RNA-Seq data comprised paired- end reads with read length of 43bp. Reads were aligned to the *Drosophila* reference genome (Ensembl BDGP6 release 84 and *Wolbachia* NCBI NC_002978.6) using tophat2 (15) (--library fr-firststrand --no-coverage-search --keep-fasta-order --segment-length 20 --segment-mismatches 1). Additionally, we estimated the tophat's insert distance (--mate-inner-dist) and standard deviation (--mate-std-dev) by aligning the first 1M reads. Aligned reads were filtered by removing non-primary alignments, unmapped reads and mates, reads not mapped in proper pairs and alignments with MAPQ <20, using samtools(Li et al., 2009) (v1.3.1; -F 0x100 -F 0x004 -F 0x008 -q 20 -f 0x002). Read counts per gene identifier were computed using htseq-count (16) (v0.6.0; --stranded=reverse -t exon --idattr=gene_id --mode=intersection-strict). Differentially expressed genes were determined using DESeq2 (17) (v1.10.1) with default parameters. Genes were deemed differentially expressed if p-value ≤ 0.05 following a Benjamini and Hochberg correction for multiple hypothesis testing (default parameter in DESeq2).

Statistical significance of differential expression gene sets. Statistical significance of overlaps of genes in two expression experiments was determined using Fisher's exact test. To account for multiple hypotheses testing, a p-value cut-off of $p \leq 1.0 \times 10^{-05}$ was used.

Quantitative real-time PCR (qPCR). Total RNA was isolated from fly heads using standard Trizol (Invitrogen) protocols. RNA samples were treated with Turbo DNase (Invitrogen) and converted to cDNA using oligod(T) primers and Superscript II reverse transcriptase (Invitrogen). Quantitative RT-PCR was performed using Power SYBR Green PCR Master Mix (ABI) in the Quant Studio 6 Flex system. Relative quantities of transcripts were determined using the relative standard curve method normalized to *Tub84B*. Primer sequences were: Atg17_F: GGGCTCCAAGTTCTATCGCA; Atg17_R: CTGATAGACGCTCGTGTTGC; Tub84B_F: TGGGCCCGTCTGGACCACAA; Tub84B_R: TCGCCGTCACCGGAGTCCAT.

Western blots. For total protein extraction (ATG8 western blots), 8 fly heads per sample were homogenized in 1X RIPA buffer (NEB 9806) with protease inhibitors (Roche, Cat# 11 836 170 001), and centrifuged for 2 min at 13,000 rpm in 40C. The resulting supernatant fraction was collected for western blot. Equal quantities of protein for each sample (as determined by the BCA Protein Assay Kit (Pierce)) were then separated on Poly-Acrylamide gels (15%, Acrylamide/Bis-Acrylamide solution, Sigma A7168, made according to manufacturers recommended protocol) and transferred to a PVDF membrane.

For insoluble ubiquitinated proteins, western blots were carried out as described in detail in (18). Briefly, 10 fly heads per sample were homogenized in Triton-X buffer (1% Triton-X, 10mM NEM, 50µm MG132, Complete Mini protease inhibitors (Roche), in PBS). After centrifugation, the supernatant was collected as the soluble fraction and used for protein quantification and loading control (Actin) gels. The insoluble pellet was re-suspended in SDS buffer (2% SDS, 10mM NEM, 50µm MG132, Complete Mini protease inhibitors (Roche), 50mM Tris pH 7.4), centrifuged, and the supernatant collected as the insoluble sample. Equal quantities of protein for each sample were then separated on 4-12% NuPage Bis-Tris gels (Invitrogen) and transferred to PVDF membranes.

Membranes were blocked in 5% BSA in Tris-buffered saline (TBS) with 0.05% Tween-20 (TBST) for 1 hour at room temperature, after which they were probed with primary antibodies diluted in 5% BSA in TBST overnight at 4°C. The following primary antibodies were used: mouse anti-poly-ubiquitinated proteins (Millipore clone FK2, 1:1000), rabbit anti-ATG8 (generated exactly as in (19), 1:1000), and mouse anti-β-

Actin (Abcam #ab1801, 1:10,000). Membranes were then probed with secondary anti-mouse antibody (Abcam #ab6789, 1:10,000) or anti-rabbit antibody (Abcam #ab6721, 1:10,000) in TBST for 1 hour at room temperature. Blots were developed using Luminata Crecsendo (Millipore) and the ImageQuant LAS 4000 system. Densitometric analysis of blot images was carried out using Fiji software (20).

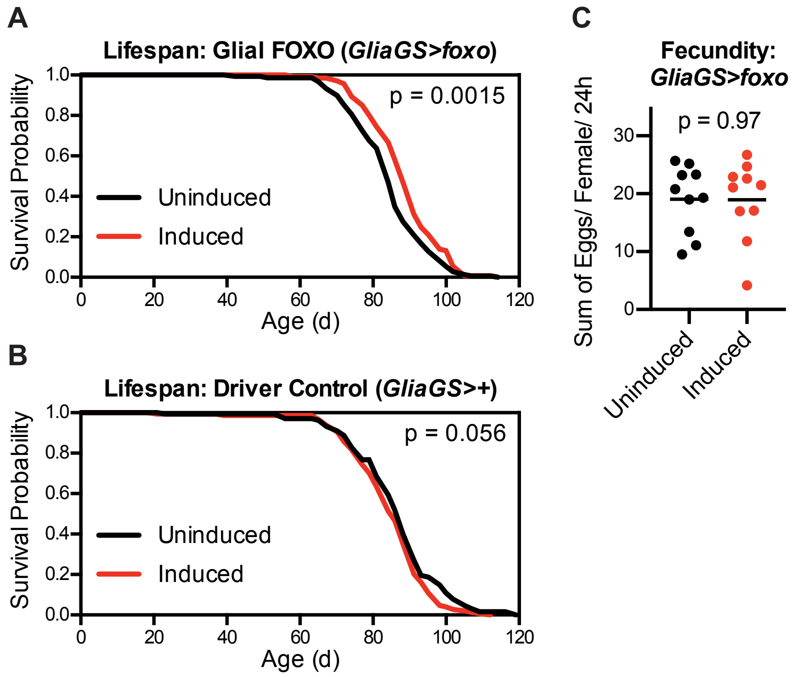

Figure S1. Over-expression of *foxo* in glia reproducibly extends lifespan without altering fecundity. Survival curves show (**A**) extended lifespan for w^{Dah}*;;GSG3285-1/UAS-foxo* flies and (**B**) no increase in lifespan for driver-control w^{Dah}*;;GSG3285-1/+* flies treated with 200μM RU-486 from 2 days of age compared to sibling flies of the same genotype treated with vehicle control food. (**C**) Fecundity assays show no significant difference in egg laying for w^{Dah}*;;GSG3285-1/UAS-foxo* flies treated with 200μM RU-486 from 2 days of age compared to sibling flies of the same genotype treated with vehicle control food. For all survival experiments, n>130 deaths counted per condition; for fecundity assays, n=10 vials of 10 females per vial per condition. p-values are from log-rank tests (**A-B**) or unpaired T-test (**C**).

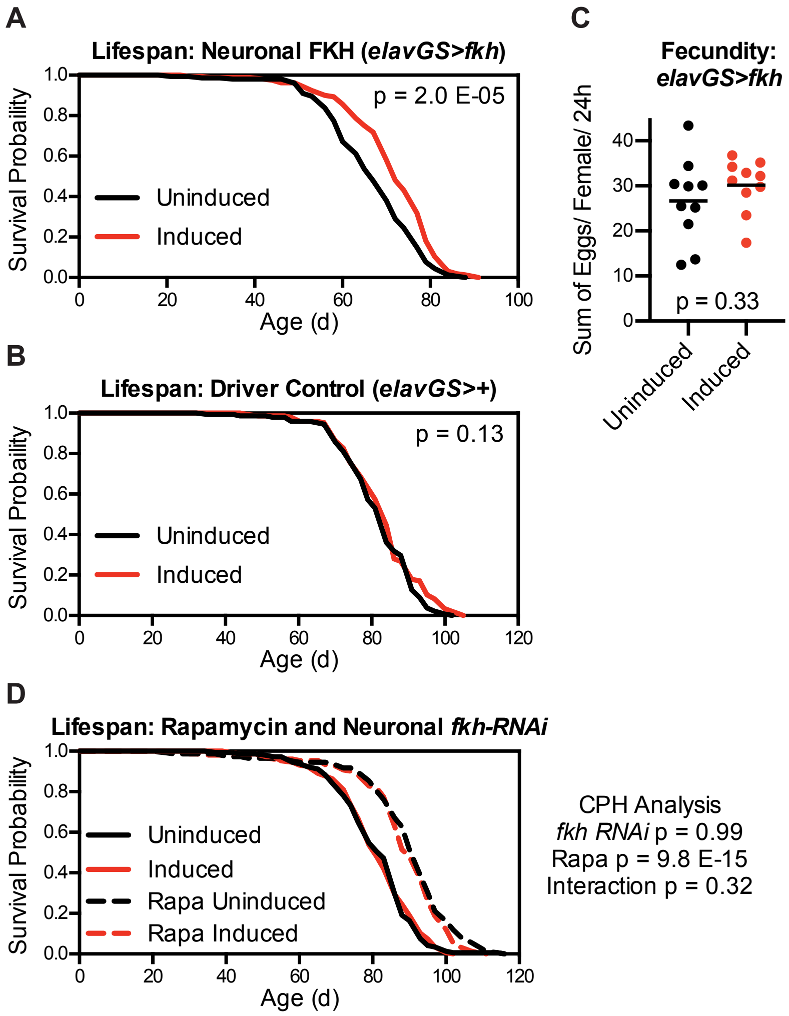

Figure S2. Over-expression of *fkh* in neurons reproducibly extends lifespan without altering fecundity, but RNAi knock-down of fkh in neurons does not interact with rapamycin treatment. (**A-B**) Survival curves show (**A**) extended lifespan for *w*^{Dah}*;UAS-fkh/+;elav-GS/+* flies and (**B**) no increase in lifespan for driver-control *w*^{Dah}*;;elav-GS/+* flies treated with 200µM RU-486 from 2 days of age compared to sibling flies of the same genotype treated with vehicle control food. (**C**) Fecundity assays show no significant difference in egg laying for *w*^{Dah}*;UAS-fkh/+;elav-GS/+* flies treated with 200µM RU-486 from 2 days of age compared to sibling flies of the same genotype treated with vehicle control food. (**D**) Survival curves show extended lifespan for *w*^{Dah}*;;UAS-fkh-RNAi/elav-GS* flies treated with 100µM rapamycin, with no effect of 200µM RU-486, from 2 days of age, with no significant interaction between *fkh-RNAi* expression and rapamycin treatment. For all survival experiments, n>140 deaths counted per condition; for fecundity assays, n=10 vials of 10 females per vial per condition. p-values are from log-rank tests (**A-B**), unpaired T-test (**C**), or Cox Proportional Hazards (**D**).

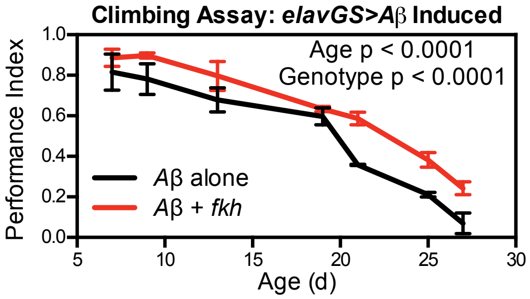

Figure S3. Over-expression of *fkh* in neurons reproducibly improves neuromuscular function in the presence of Aβ. Climbing assay results show improved neuromuscular function in induced (200μM RU-486) *w*^{1118}*;UAS-Aβ*$_{Arc}$*/UAS-fkh;elav-GS/+* flies compared to induced *w*^{1118}*;UAS-Aβ*$_{Arc}$*/+;elav-GS/+* flies. n=2-3 vials of 15 flies per vial per condition; p-values are from 2-way ANOVA.

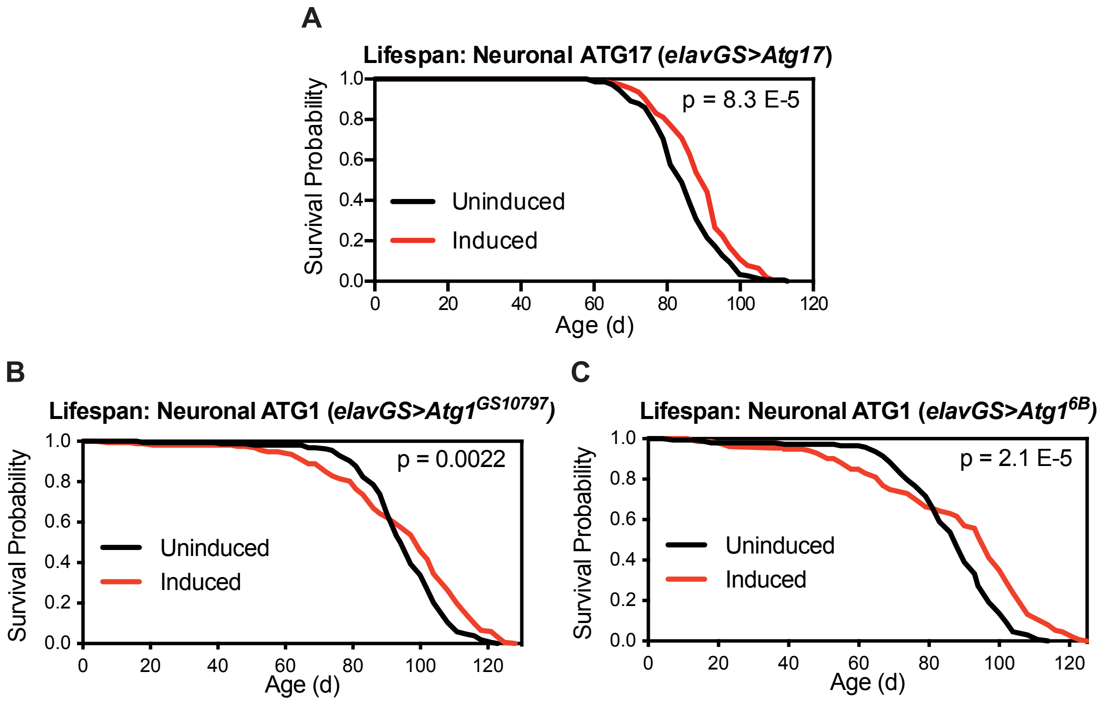

Figure S4. Over-expression of *Atg17* or *Atg1* in neurons reproducibly extends lifespan. Survival curves show extended lifespan for (**A**) w^{Dah}*;UAS-Atg17/+;elav-GS/+* flies, (**B**) w^{Dah}*;;elav-GS/UAS-Atg1*^{GS10797} flies, with 3.1% increase in median and 4.2% increase in maximum lifespan, and (**C**) w^{Dah}*;;elav-GS/UAS-Atg1*^{6B} flies, with 9.8% increase in median and 12.7% increase in maximum lifespan, when treated with 200μM RU-486 from 2 days of age compared to sibling flies of the same genotype treated with vehicle control food. n>135 deaths counted per condition; p-values are from log-rank tests.

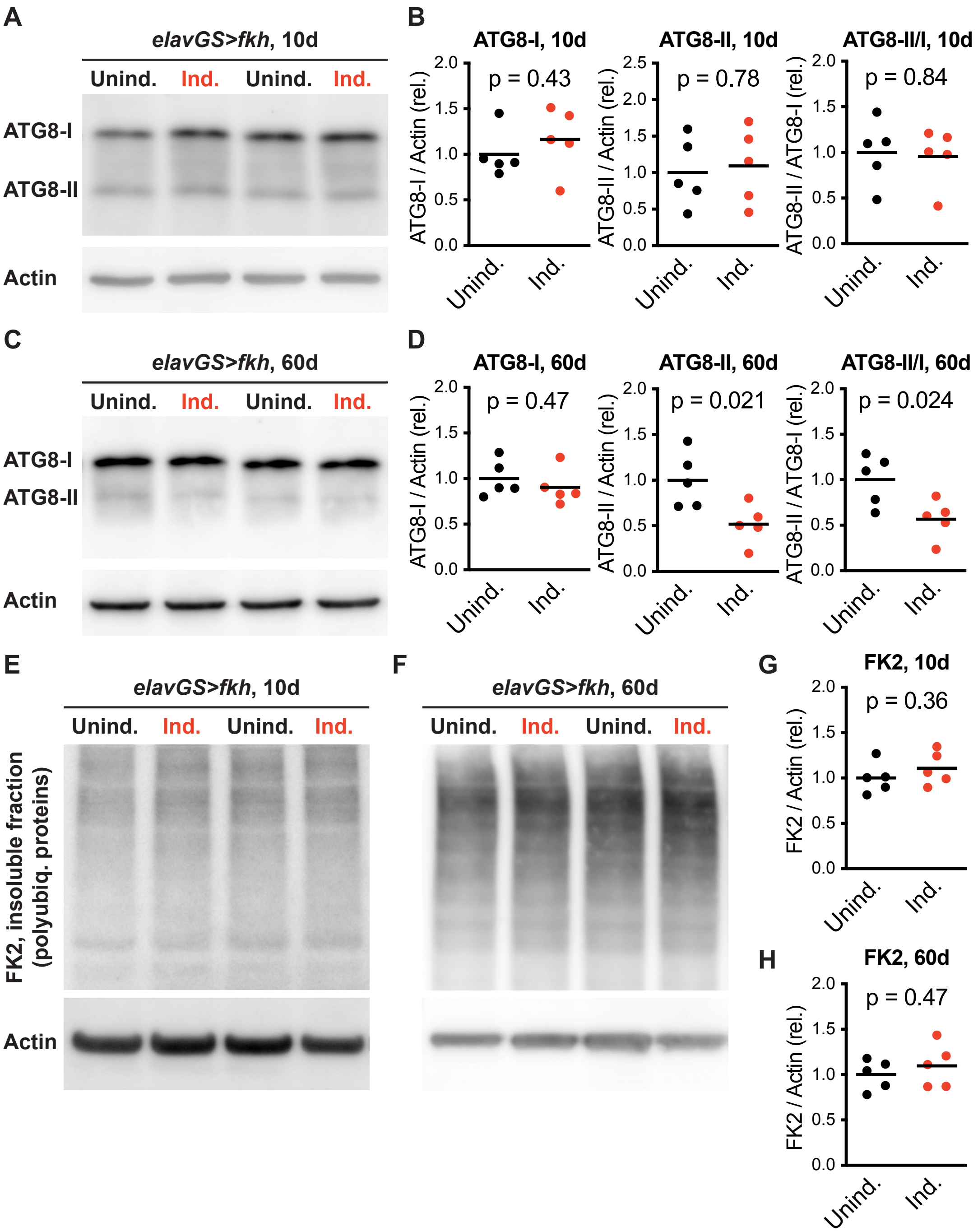

Figure S5. Over-expression of *fkh* in neurons alters ATG8-II levels in aged flies, with no change in insoluble ubiquitinated protein levels. Western blots were performed from head extracts of 10-day-old (**A-B,E,G**) or 60-day-old (**C-D,F,H**) w^{Dah}*;UAS-fkh/+;elav-GS/+* flies treated with 200μM RU-486 from 2 days of age compared to sibling flies of the same genotype treated with vehicle control food. Blots show no significant differences for (**A-B**) ATG8-I or ATG8-II in 10-day-old flies, with (**C-D**) significantly lower levels of ATG8-II in *fkh* over-expressing 60-day-old flies. (**E-H**) Levels of FK2-positive insoluble ubiquitinated proteins were not significantly different with *fkh* over-expression in either 10-day-old or 60-day-old flies. n=5 replicates of 8-10 heads per sample per condition; p-values are from unpaired T-tests.

Table S1. Raw log2-Fold-Change and adjusted p-values from the top *fkh*-dependent genes responding to reduced IIS, listed in the main manuscript in **Figure 4**.

FlyBase Gene ID	Gene Name	log2FC[a] (IIS effect)	adj_p[b] (IIS effect)	log2FC[a] (FKH effect)	adj_p[b] (FKH effect)
FBgn0052512	CG32512	1.028	5.07E-67	0.695	1.91E-26
FBgn0013984	InR	2.831	<1.0E-220	0.374	3.46E-10
FBgn0039681	CG7582	0.915	2.98E-33	0.550	1.36E-09
FBgn0261560	Thor	0.558	3.57E-37	0.327	6.83E-09
FBgn0026878	CG4325	1.115	1.39E-27	0.702	8.91E-09
FBgn0030326	CG2444	1.247	2.51E-24	0.967	2.06E-07
FBgn0001090	bnb	0.504	1.36E-23	0.269	1.65E-05
FBgn0033257	CG8713	1.094	2.67E-24	0.453	5.78E-03
FBgn0027945	ppl	0.440	6.57E-20	0.662	9.02E-35
FBgn0001257	ImpL2	0.520	4.87E-18	0.430	4.20E-09
FBgn0016123	Aph-4	0.506	2.38E-15	0.463	2.14E-11
FBgn0033483	egr	0.583	4.15E-15	0.434	5.70E-06
FBgn0025631	moody	0.424	1.38E-14	0.368	9.05E-09
FBgn0086443	Aats-asn	0.428	8.43E-14	0.569	3.86E-20
FBgn0000071	Ama	0.770	1.50E-13	0.915	1.93E-05
FBgn0037363	Atg17	0.366	8.83E-13	0.249	8.25E-04
FBgn0032400	CG6770	0.454	1.36E-12	0.221	7.92E-04
FBgn0038197	foxo	0.443	3.18E-12	0.418	2.40E-09
FBgn0262582	cic	0.350	1.48E-10	0.641	1.32E-29
FBgn0034717	CG5819	0.411	6.51E-10	0.368	4.54E-06
FBgn0039298	to	-0.385	2.89E-07	-0.989	1.19E-55
FBgn0036698	CG7724	-0.449	2.52E-07	-0.453	2.12E-05
FBgn0039178	CG6356	-0.379	5.29E-08	-0.281	4.02E-04
FBgn0038799	CG4288	-0.734	1.48E-08	-0.687	2.09E-03
FBgn0033065	Cyp6w1	-0.413	8.91E-09	-0.838	5.88E-58
FBgn0039294	Cad96Cb	-0.596	7.72E-09	-0.992	5.08E-16
FBgn0083956	CG34120	-0.306	6.24E-09	-0.625	3.86E-35
FBgn0026418	Hsc70Cb	-0.293	4.50E-09	-0.158	3.55E-02
FBgn0010053	Jheh1	-0.332	4.02E-09	-0.496	2.82E-20
FBgn0024251	bbx	-0.714	6.52E-10	-0.465	2.85E-02
FBgn0015569	alpha-Est10	-0.436	4.13E-11	-0.550	7.86E-12
FBgn0039754	CG9747	-0.544	3.33E-12	-0.720	7.19E-36
FBgn0029898	CG14439	-0.424	2.52E-12	-0.324	5.18E-05
FBgn0015623	Cpr	-0.312	1.88E-14	-0.429	3.21E-19
FBgn0019643	Dat	-0.483	9.59E-17	-0.574	1.18E-20
FBgn0034501	CG13868	-0.426	1.54E-18	-0.386	1.58E-06
FBgn0011581	Ms	-0.459	1.36E-22	-0.265	1.02E-05
FBgn0020513	ade5	-0.497	1.97E-23	-0.446	1.15E-08
FBgn0026268	antdh	-1.185	3.05E-35	-1.597	4.05E-57
FBgn0000473	Cyp6a2	-0.520	1.40E-22	-0.876	2.17E-39

a: log2FC = $\log_2$ Fold Change
b: adj_p = False Discovery Rate (FDR) corrected p-values

SI References

1. T. Osterwalder, K. S. Yoon, B. H. White, H. Keshishian, A conditional tissue-specific transgene expression system using inducible GAL4. *Proc. Natl. Acad. Sci. U. S. A.* **98**, 12596–12601 (2001).
2. M. Latouche, *et al.*, A conditional pan-neuronal Drosophila model of spinocerebellar ataxia 7 with a reversible adult phenotype suitable for identifying modifier genes. *J. Neurosci.* **27**, 2483–2492 (2007).
3. L. Nicholson, *et al.*, Spatial and temporal control of gene expression in drosophila using the inducible geneSwitch GAL4 system. I. Screen for larval nervous system drivers. *Genetics* **178**, 215–234 (2008).
4. D. Yamazaki, *et al.*, Glial dysfunction causes age-related memory impairment in Drosophila. *Neuron* **84**, 753–763 (2014).
5. H. Tricoire, *et al.*, The steroid hormone receptor EcR finely modulates Drosophila lifespan during adulthood in a sex-specific manner. *Mech. Ageing Dev.* **130**, 547–552 (2009).
6. E. Bolukbasi, *et al.*, Intestinal Fork Head Regulates Nutrient Absorption and Promotes Longevity. *Cell Rep.* **21**, 641–653 (2017).
7. M. A. Jünger, *et al.*, The Drosophila Forkhead transcription factor FOXO mediates the reduction in cell number associated with reduced insulin signaling. *J. Biol.* **2**, 20 (2003).
8. C. Slack, M. E. Giannakou, A. Foley, M. Goss, L. Partridge, dFOXO-independent effects of reduced insulin-like signaling in Drosophila. *Aging Cell* **10**, 735–748 (2011).
9. D. C. Crowther, *et al.*, Intraneuronal Aβ, non-amyloid aggregates and neurodegeneration in a Drosophila model of Alzheimer's disease. *Neuroscience* **132**, 123–135 (2005).
10. J. Y. Roignant, *et al.*, Absence of transitive and systemic pathways allows cell-specific and isoform-specific RNAi in Drosophila. *Rna* **9**, 299–308 (2003).
11. P. Nagy, *et al.*, Atg17/FIP200 localizes to perilysosomal Ref(2)P aggregates and promotes autophagy by activation of Atg1 in Drosophila. *Autophagy* **10**, 453–467 (2014).
12. O. Sofola, *et al.*, Inhibition of GSK-3 ameliorates Aβ pathology in an adult-onset Drosophila model of Alzheimer's disease. *PLoS Genet.* **6**, e1001087 (2010).
13. K. Davie, *et al.*, A Single-Cell Transcriptome Atlas of the Aging Drosophila Brain. *Cell* **174**, 982-998.e20 (2018).
14. E. W. Abrams, W. K. Mihoulides, D. J. Andrew, Fork head and Sage maintain a uniform and patent salivary gland lumen through regulation of two downstream target genes, PH4αSG1 and PH4αSG2. *Development* **133**, 3517–3527 (2006).
15. C. Trapnell, L. Pachter, S. L. Salzberg, TopHat: Discovering splice junctions with RNA-Seq. *Bioinformatics* **25**, 1105–1111 (2009).
16. S. Anders, P. T. Pyl, W. Huber, HTSeq-A Python framework to work with high-throughput sequencing data. *Bioinformatics* **31**, 166–169 (2015).
17. M. I. Love, W. Huber, S. Anders, Moderated estimation of fold change and dispersion for RNA-seq data with DESeq2. *Genome Biol.* **15**, 1–21 (2014).
18. R. C. Cumming, A. Simonsen, K. D. Finley, Quantitative analysis of autophagic activity in Drosophila neural tissues by measuring the turnover rates of pathway substrates. *Methods Enzymol.* **451**, 639–651 (2009).
19. J. M. I. Barth, J. Szabad, E. Hafen, K. Köhler, Autophagy in Drosophila ovaries is induced by starvation and is required for oogenesis. *Cell Death Differ.* **18**, 915–924 (2011).
20. J. Schindelin, *et al.*, Fiji: An open-source platform for biological-image analysis. *Nat. Methods* **9**, 676–682 (2012).
